# A “well-kept secret”: facilitators and barriers to occupational therapy referral as a component of supportive cancer care in the United States

**DOI:** 10.3389/fmed.2026.1799055

**Published:** 2026-08-05

**Authors:** Khawla Loubani, Katie M. Polo, Morgan Jones, Korrin Schalhamer, Olivia Witteborg, Alexandria Gilley, Cara Murphy, Taylor Brown, Rachel Graves

**Affiliations:** 1 Department of Occupational Therapy, Faculty of Health Sciences, Ben-Gurion University of the Negev, Be'er Sheva, Israel; 2 School of Occupational Therapy, University of Indianapolis, Indianapolis, IN, United States

**Keywords:** occupational therapy, cancer rehabilitation, qualitative research, referral pathways, supportive cancer care, cancer survivorship (public health)

## Abstract

**Introduction:**

People living with and beyond cancer often experience persistent physical, cognitive, psychosocial, and functional challenges that require supportive rehabilitation across the cancer continuum. Occupational therapy (OT) can address these needs by supporting daily functioning, participation, self-management, and quality of life; however, OT remains underutilized in cancer care. Because access to OT often depends on referral decisions made by oncology professionals, understanding how referral sources perceive OT and navigate referral processes is essential for improving timely access to supportive cancer-care services. This study explored cancer-care professionals’ perspectives on barriers, facilitators, and solutions to strengthen OT referral as part of supportive cancer-care delivery for persons living with and beyond cancer.

**Methods:**

This grounded-theory-informed, qualitative, multiple-case study included four videoconference focus groups and 17 participants. Data were analyzed using Corbin and Strauss’s three-stage approach. A final logic diagram was developed to organize multilevel barriers and facilitators and to generate theoretical propositions regarding how referral sources’ knowledge, OT visibility, organizational processes, and system-level constraints shape OT referral decision-making.

**Results:**

Three main themes emerged: (1) *Facilitators* included referral sources’ knowledge of OT’s scope and organizational processes, and the integration of OT into cancer-care protocols; (2) *Barriers* included insufficient knowledge and awareness of OT’s role, low prioritization of functional concerns, limited OT availability in cancer settings, and system- and patient-level obstacles, such as insurance costs and clinic workflow constraints; and (3) *Solutions* emphasized increasing OT presence in infusion clinics and community settings, enhancing interdisciplinary collaboration, and clarifying OT’s unique contributions through targeted education and marketing. These findings highlight missed opportunities for timely, coordinated supportive rehabilitation within cancer care.

**Conclusion:**

Despite recognition of OT’s potential value, multiple multilevel barriers limit access to OT services for cancer survivors in the US. Addressing these barriers requires coordinated strategies, including embedding OT practitioners within interdisciplinary cancer-care teams, expanding service delivery models, and improving awareness among referral sources. These strategies may support earlier identification of functional needs and improve access to timely, coordinated supportive rehabilitation across the cancer continuum.

## 1 Introduction

Despite advances in cancer treatment leading to improved survival rates, people living with and beyond cancer (PLWBC) face persistent physical, cognitive, and psychosocial challenges that significantly impact daily function and quality of life (1–3). While medical oncology focuses on disease eradication and survival, the functional consequences of cancer and its treatments, including fatigue, neuropathy, cognitive changes, and lymphedema, often remain inadequately addressed. Supportive rehabilitation services, including occupational therapy (OT), are uniquely positioned to address these needs throughout the cancer continuum (2, 4). However, despite growing evidence supporting OT’s effectiveness in cancer rehabilitation, these services remain significantly underutilized (2–8).

There are many gaps in the literature regarding the referral process to OT in cancer care. Few studies have examined how referral processes influence access to supportive rehabilitation services within oncology care, particularly from the perspective of the referral sources, who shape care delivery (9). From a health professions education (HPE) and interprofessional collaboration perspective, referral behavior is shaped not only by clinical need but also by how professional roles are recognized, understood, and negotiated within teams. Interprofessional identity and role clarification are important components of collaborative practice, particularly when team members must understand each profession’s distinct contribution to patient care. Recent evidence also suggests that interprofessional education can improve healthcare professionals’ understanding of OT roles and responsibilities. These concepts are directly relevant to cancer care, where limited recognition of OT’s role may contribute to delayed or absent referral despite patients’ functional needs (10–13).

Considering the implications of delayed or missed rehabilitation referrals on functional outcomes and quality of life, understanding referral sources’ knowledge, attitudes, and perceived barriers is essential for developing targeted interventions to improve PLWBC’s access to OT services (9). These gaps are particularly salient, given referral sources’ central role in identifying rehabilitation needs and initiating access to OT services. Recent reviews continue to note that limited awareness of OT’s role among healthcare professionals and patients contributes to the underutilization of OT services in oncology, further supporting the need to examine referral sources’ knowledge and referral decision-making (7, 14).

The healthcare system structure significantly influences access to rehabilitation services and referral patterns. An exploration of perceived facilitators and barriers to OT referral by Israeli cancer referral sources revealed that collaboration and communication with OT practitioners and awareness of OT services among cancer-care referral sources facilitated referrals (9). The study also revealed barriers: the referral sources’ lack of knowledge about OT’s role in cancer care, difficulty tying cancer side effects to daily performance and functional issues indicating the need for referral, inability to understand how and which patients to refer, bureaucracy, absence of OT practitioners in cancer-care institutions and nearby communities, and lack of infrastructure to facilitate referrals (9, 15).

Although the study by Loubani et al. (9) was the first of its kind to explore factors related to OT referral in cancer care, these findings were specific to Israel. Little is known about factors related to cancer-care referrals in the United States (16). Israel’s universal healthcare system provides centralized referral pathways with minimal out-of-pocket costs for OT services (17). In contrast, the US system’s fragmented insurance landscape creates variable access depending on coverage type, state regulations, and provider networks (18). These structural differences may produce distinct barriers and facilitators to OT referral. Therefore, examining the US context is essential for developing contextually appropriate strategies to improve OT access in US cancer care.

This study is Phase II of a binational collaboration among OT researchers from Israel and the United States. It aims to examine referral sources’ perspectives on OT’s role and identify barriers and facilitators influencing OT referral in the United States. Such perspectives are critical for understanding system-level factors because referral practices fundamentally shape access to supportive cancer care across the continuum in the US. The specific aims of this study were to (a) explore US cancer-care professionals’ (hereafter referred to as *referral sources*) knowledge and perspectives regarding the role and value of OT services for PLWBC across the cancer continuum, (b) identify facilitators and barriers to OT referrals from the perspective of US referral sources, and (c) elicit recommendations from referral sources for optimizing OT integration and access within supportive cancer-care settings in the US.

## 2 Methods

### 2.1 Research design

This study used a grounded theory approach with a multiple explanatory case-study design and a semi-structured interview guide (19, 20). This approach was selected because it enables the development of a theory grounded in participants’ lived experiences, making it ideal for exploring a phenomenon (i.e., OT referral patterns in cancer care) that lacks comprehensive theoretical understanding. The multiple-case-study design allows case comparison across practice settings and professional roles.

### 2.2 Participants and recruitment

For this study, purposive and snowball sampling strategies were utilized to intentionally select cancer care providers based on their oncology clinical expertise. This approach ensured that the qualitative insights gathered were derived from participants uniquely qualified to address the specific research objectives (10). Professional contacts of the researchers and a list of open-access oncology contacts from major cancer institutes nationwide were used to inform potential participants. Recruitment of participants occurred via email over a five-month period. Participants were eligible if, at the time of the study, they were practicing in oncology or cancer care in the United States and worked in settings such as hospitals, community cancer centers, or cancer clinics. Eligible participants also had to be working in a professional role that could involve identifying supportive care needs or referring patients to occupational therapy services. Eligible professional roles included healthcare professionals working in oncology or cancer care (e.g., nurses, physicians/oncologists, social workers, and physician assistants) across hospitals, community cancer centers, and cancer clinics. No minimum number of years of experience in oncology or cancer care was required, as the study aimed to capture the perspectives of frontline professionals currently involved in cancer care and referral-related decision-making in the US. Four videoconference–based focus groups were conducted with four to six participants to balance depth of exploration with participants’ time constraints while allowing for emergent themes. The focus groups were planned to support thematic identification and cross-case analysis (10); each lasted approximately 45 min. In total, 17 participants were recruited (two oncologists, one breast surgeon, five nurse navigators, six nurses, and three social workers) from several states: nine (52.9%) from Indiana, six (35.3%) from Texas, one (5.9%) from Florida, and one (5.9%) from Illinois. Their mean age was 48.33 years (*SD* = 10.91); two men and 15 women.

Notably, despite targeted recruitment efforts, physician participation remained limited (*n* = 3, 17.6%). This result reflects well-documented challenges in recruiting physicians for qualitative research due to their time constraints and competing clinical demands. To address this gap, the sample included nurse navigators and advanced practice nurses involved in referral decision-making.

### 2.3 Data collection

Two experienced qualitative researchers supervised and supported seven OT doctoral student researchers (OTD) through data collection and analysis. Additionally, the OTDs received training in focus-group facilitation, field-note documentation, and member-checking under the oversight of senior researchers and an external qualitative research expert. The research team had no prior relationships with participants before the focus groups. Potential participants were contacted via email, given information about the study through a Qualtrics survey, and asked to complete a demographic questionnaire (Supplementary material 1). The demographic questionnaire collected information on participants’ age, gender, education level, profession, specialty, state of practice, practice setting, years working in oncology, workplace/unit/department, job title, active referral to OT, ability to collaborate directly with occupational therapists, and frequency of direct collaboration with occupational therapists.

Three researchers, including one interviewer and two field-notetakers, facilitated each focus group. The groups included participants from diverse professional backgrounds to capture interdisciplinary perspectives on referral processes. Demographic and qualitative data were collected using a demographic questionnaire and semi-structured interview guide with open-ended questions adapted from Phase I (9). The interview guide (Supplementary material 2) included comparable core domains, with minor adaptations to fit the United States cancer-care context. The Phase I interview guide was originally developed by doctoral-level occupational therapy researchers with expertise in oncology rehabilitation, qualitative research, and occupational therapy service delivery. The questions were informed by the study aims and were designed to elicit participants’ perspectives on occupational therapy referral processes, perceived rehabilitation needs, barriers and facilitators to referral, and strategies to improve access to occupational therapy services. Before its use in Phase I, the guide was reviewed and revised with input from external PhD-level occupational therapy researchers with extensive qualitative research experience. The questions explored referral processes across the cancer continuum, perceptions of OT’s role, perceived rehabilitation needs, barriers to referral, and strategies to optimize access to services. All focus groups were conducted via Google Meet, audio-recorded, and transcribed verbatim.

### 2.4 Data analysis

Descriptive demographic data were analyzed using IBM SPSS (Version 27). Two research team members reviewed each focus group transcript for accuracy. Qualitative data were analyzed using a grounded theory approach, following the three stages of Corbin and Strauss (21):

(1) Open Coding. Two team members independently reviewed each transcript line-by-line to identify initial concepts and categories. The team met regularly to compare codes, discuss discrepancies, and develop a preliminary codebook with operational definitions.
(2) Axial Coding. Seven OTD researchers were involved in axial coding under the supervision of two senior qualitative researchers. During this stage, relationships between categories were examined to identify patterns and connections. This stage involved constant comparison across transcripts and focus groups to refine categories and establish subcategories.
(3) Selective Coding. The team integrated categories into overarching themes and developed theoretical propositions explaining relationships between facilitators and barriers.

Analytic memoing was conducted throughout the analysis, to document decisions and theoretical insights. Data saturation was reached during analysis of the third transcript; analysis of the fourth focus group confirmed the established themes without revealing new categories. Coding decisions were discussed until consensus was achieved.

### 2.5 Reflexivity and positionality

The research team included seven OTD student researchers and two senior occupational therapy researchers with expertise in oncology rehabilitation and qualitative research. The team’s occupational therapy background provided disciplinary insight into OT’s potential role in cancer care, but also required reflexive attention to assumptions regarding the value, visibility, and underuse of OT services. Throughout data collection and analysis, the research team engaged in reflexive discussions regarding how professional identities, prior clinical and research experiences, and power dynamics between student researchers, senior researchers, and participants might influence questioning, interpretation, and theme development. The senior researchers, first and last authors, who had led the Phase I Israeli study, did not actively code the data but supported analytic discussions, reviewed emerging categories, and helped ensure methodological rigor.

### 2.6 Trustworthiness and credibility

The audit trail was maintained from the beginning of the study and included index tracking of initial and final coding, as well as notes on decision-making to increase dependability and confirmability. Collectively, the research teams engaged in continual reflexive thinking by considering and challenging how biases and experiences might influence data interpretation (19), including thinking about one’s thinking, observing emotions and thoughts, considering role boundaries and power dynamics in the researchers’ relationships, and exploring perceptual experiences. Additionally, the first and last authors, experienced qualitative researchers who analyzed the first Israeli study, did not actively code data for this study. They supervised the other researchers during the analysis to ensure that findings from the first study would not influence or bias findings in the second study. To ensure triangulation and establish credibility, multiple data sources were collected, including video recordings, transcriptions, and field notes from focus group discussions (19). An external OT researcher with expertise in qualitative data analysis conducted an external audit to confirm logical reasoning and strengthen dependability. To confirm and support credibility, member checking was conducted at the end of each focus group. Field-notetakers provided a summary of the discussion, and participants were invited to clarify, correct, or add information before the session ended (19).

### 2.7 Ethical considerations

This exempt review was conducted according to the Helsinki Declaration, based on the Phase I study (9). The University of Indianapolis Institutional Review Board approved the current study (Phase II). All participants provided informed consent upon enrolling in the study. Furthermore, at the beginning of each focus group, researchers explained the study’s aims and assured participants of their right to decline any questions or withdraw from the study at any time. All transcripts were de-identified to ensure the participants’ confidentiality.

## 3 Results

Table 1 presents the participants’ demographic data. Four participants had missing demographic information. Nearly half of the participants (47.1%) actively referred patients to OT services.

**Table 1:** Participants’ sociodemographic characteristics.

Participant	Age (yr)^a^	Highest degree^b^	Profession^c^	Title/Department	Practice setting^d^	Years in cancer care^e^	Collaborates with OT^f^	Actively refers to OT^g^
1	47	MD	Med	Breast surgeon	Academy	15	Yes	Yes
2	66	MA/S	Nu	Clinical research coordinator	Community	23	No	No
3	60	BA/S	Nu	Thoracic oncology nurse navigator	Other	40	Yes	Yes
4	65	MA/S	Nu	Registered nurse abstractor	Other	40	No	No
5	35	AA/S	Nu	Nursing services manager	Community	4	No	Yes
6	54	AA/S	Nu	Primary clinic nurse	Community	24	No	Yes
7	45	BA/S	Nu	Clinic nurse	Community	1	No	No
8	NR	NR	Med	Oncologist	NR	NR	NR	NR
9	NR	NR	Nu	NR	NR	NR	NR	NR
10	30	MA/S	SW	Oncology social worker	Hospital	4	No	Yes
11	56	MA/S	SW	Social worker	Hospital	7	No	No
12	38	MD	Med	Medical oncologist	Hospital	7	Yes	Yes
13	36	MA/S	SW	Program manager	Community	8	No	No
14	54	BA/S	Nu	NR	NR	NR	NR	NR
15	49	BA/S	Nu	Nurse navigator	Community	15	No	Yes
16	43	AA/S	Nu	Breast navigator	Hospital	10	Yes	Yes
17	47	MA/S	Nu	NR	NR	NR	NR	NR

*N* = 17; four participants had missing data (we attempted to contact them after the interviews to complete the missing information but were unable to reach them); all except Participants 8 and 12 were women. Med, medicine; Nu, nursing; SW, social work, OT, occupational therapy. AA/S, associates of arts/science; BA/S, bachelor of arts/science; MA, master of arts/science; NR, not reported.

^a^*M* = 48.33 (SD = 10.91); ^b^BA/S: 4 (23.5%), MA/S: 6 (35.3%), MD: 2 (11.8%), AA/S: 3 (17.6%); NR: 2 (11.8%); ^c^Nu: 11 (64.7%), SW: 3 (17.6%), Med: 3 (17.6%); ^d^Hospital: 4 (23.5%), Community: 6 (35.3%), Academy: 1 (5.9%), Other: 2 (11.8%), NR: 4 (23.5%); ^e^Mdn = 10.0 years (interquartile range = 7–23); NR: 4 (23.5%); ^f^Yes: 4 (23.5%), No: 9 (52.9%), NR: 4 (23.5%); ^g^Yes: 8 (47.1%), No: 5 (29.4%), NR: 4 (23.5%).

Analysis of the focus-group discussions revealed complex, multilevel factors influencing OT referrals in US cancer care. Three main themes emerged: (1) Facilitators for OT referral in cancer care included the subthemes of *referral sources’ knowledge* and *processes that integrate OT*; (2) Barriers to OT referral included those operating at the individual-provider, organization/clinic, system, and patient levels; and (3) Suggestions to support OT referrals included three subthemes: (a) bolstering OT presence in other settings, including infusion clinics, (b) increasing interdisciplinary care presence, and (c) clarifying OT’s role in cancer care. The themes are interconnected, with barriers often representing the inverse of facilitators.

### 3.1 Theme 1: Facilitators

Facilitators for OT referral reflected organizational and professional conditions that enabled access to supportive rehabilitation within cancer care. As shown in Table 2, these included the referral sources’ knowledge of OT, OT’s scope of practice, and processes for integrating OT into routine care. The participants’ knowledge of OT ranged along a continuum from limited, impairment-focused understanding to comprehensive, occupation-centered perspectives.

**Table 2:** Illustrative quotes supporting facilitators for occupational therapy referral within cancer care.

Subtheme	Facilitator	Illustrative quote
Referral sources’ knowledge of occupational therapy (OT)	Impairment-focused understanding of OT	“Having patients post chemotherapy, … a lot of weakness afterwards and need to regain that strength, … she had not been as mobile as she should have been.”
	Occupation-centered understanding of OT	“OT can help with a lot of different things, … dressing, moving around, self-care. … The goal is to make people functional, … despite limitations.”
	Personal or family exposure to OT	“My son was in OT when he was small, … then, my sister is in OT school. … That’s what I know.”
Processes that integrate OT	Automatic referral embedded in care pathways	“It gets written into our ERAS. protocol, … OT reduced the need for narcotics. … We refer all post-surgical patients and then again during survivorship, if needed.”

ERAS, Enhanced Recovery After Surgery.

Limited knowledge was associated with viewing OT primarily in relation to discrete physical impairments, whereas comprehensive knowledge reflected an understanding of OT’s role in supporting daily functioning, independence, and participation. Referral sources with broader, occupation-centered knowledge or personal/family exposure to OT services were more likely to identify appropriate referral opportunities.

Organizational processes that embedded OT within cancer-care pathways also facilitated referral. These included formal integration of OT into clinical protocols and automatic referrals after specific cancer-related surgeries, which normalized OT involvement and supported early rehabilitation.

### 3.2 Theme 2: Barriers

As depicted by Table 3’s representative quotations, participants described barriers to OT referral as operating across multiple, interrelated levels, including individual-provider, organization/clinic, system, and patient. Insufficient knowledge of OT’s scope of practice and competing clinic priorities limited referrals at the individual-provider level. Although participants acknowledged the potential benefits of OT for PLWBC, their uncertainty about when and how to refer often resulted in missed opportunities, particularly during active cancer treatment, when they prioritize disease management. Referrals were also described as highly dependent on physician initiation, restricting access in the absence of explicit endorsement. At the organization/clinic level, limited availability of OT services and high clinical workload within busy oncology settings reduced attention to functional concerns. System-level barriers included complex, unpredictable insurance coverage and reimbursement processes that discouraged referrals to outpatient therapy. At the patient level, time constraints, financial burdens, and competing treatment demands limited engagement with additional rehabilitation services.

**Table 3:** Illustrative quotes supporting barriers to occupational therapy referral across the cancer-care continuum.

Level	Barrier	Illustrative quote
Individual provider	Limited knowledge and awareness of OT	“I think OT is a well-kept secret. … Everybody knows what PT is, … unless we have something really specific, if somehow we happen to get referred to an OT, we do not know half of what you all do. You have a lot to offer, but nobody knows you are around.”
	Competing clinical priorities	“[Oncologists] want to focus on the treatment right then. … It’s getting them through the treatment, and so they will think about that after.”
	Dependence on physician initiation	“You cannot get an OT referral unless the doctor does it. So, OT practitioners are always relying on a physician to make that referral.”
Organization/clinic	Limited availability of OT services	“The biggest one we see is [OT] not being available. … There’s not a lot of OT practitioners available in home health, so a lot of times we’ll write a physical therapy eval for OT needs.”
	Busy clinic environments	“Clinics are busy. Physicians are having trouble seeing a lot of patients. … Unless they have been tuned in, the nurses do not think of it.”
System	Insurance complexity and unpredictable costs	“The pricing scheme is very opaque, … no information about what the insurance will be until six months later. … Oftentimes, there’s a lot of surprise billing.”
Patient	Competing demands during treatment	“When the patient is in the throes of treatment, it’s hard for them to think about adding an additional appointment.”

### 3.3 Theme 3: Suggestions

As illustrated by example quotations in Table 4, the participants’ suggestions to reduce patient burden while improving coordinated supportive care encompassed three subthemes: strengthening OT presence within existing and emerging cancer-care settings, enhancing interdisciplinary collaboration, and clarifying OT’s role in cancer care. Participants emphasized the importance of expanding OT services across care settings, including outpatient, home health, and infusion clinics, to facilitate timely and accessible referrals. They viewed increased interdisciplinary collaboration and proactive communication with referral sources as essential for improving coordination and continuity of care. They also highlighted the need for clearer articulation of OT’s role through targeted education and practical examples illustrating appropriate referral scenarios.

**Table 4:** Participants’ suggestions for improving referrals to OT and supportive quotes.

Suggestion	Participant’s supportive quote	
Bolstering presence in current and new cancer practice settings	Outpatient, home health	“If the patient is homebound, however, and they are only leaving for appointments, and PT/OT can visit them at home, that’s much more appealing to the patient than going to another appointment. … If that’s something that we can offer them, that seems to be a better option for the patients that are currently undergoing treatment.”
	Infusion clinic	“Being able to do … some during chemotherapy, possibly while they are sitting there, trapped availability, for those kinds of things would be convenient. … That would limit the lack of time issue on their part too, if they complete a lot of their treatment while they are still in the hospital.”
Increasing interdisciplinary care collaborations	Implement new protocols in the infusion clinic to assess the concurrent need for OT during patient treatments	“We recently have really tried to make use of our partnership with one of our local groups that we refer to a couple times in this conversation. … What I have agreed to do … is to have them actually, physically come into the clinic in our infusion room and do some assessing, talking to the patients directly, … kind of with an agreement that if they see something, then they’ll say something to us, and we’ll refer based on that, … kind of an extra set of professional eyes. They know what they are looking for, or what they are listening for in conversations.”
	Streamline therapy services to lessen patient burden	“For those maybe still trying to work [and] are not considered homebound, … doing OT after they have finished treatment or, if they have gotten away from treatment and their strength has not returned, then it’s something that they are more apt to consider.”
	Increase collaborations with survivorship navigators	“The navigator plays a huge role in that [referral process] because … they get to spend more time with the patient than … anybody else does. And so they are picking up on these subtleties. So, yeah, that’s going to be … your best way to make sure that there’s more referrals.”
Steps to clarify OT’s role	Use evidence-based OT case studies in cancer care	“Case studies, where it’s outside the box, and we know the general overview of what you do. But there’s a lot more to it, and when we see that, then it helps to think, ‘Oh my gosh. Yeah, this patient can use that’.”
	Clearly market the OT profession	“Unfortunately, I think it’s that you all do not market yourselves very well. … Again, just clarification of roles, … maybe a presentation of services that are available or like, maybe a tips and trick sheet that allows you to have a quick reference. … That would be handy for the physicians, especially because when you are placing an order for something, they need to know where to be able to do this. … [That] would make it much simpler for the physician or nurse practitioner to be able to get somebody where they need to be. Then the navigator can discuss it with the patient and intervene on their behalf, with the physician saying, ‘You know, there is OT that can take care of this particular issue’.”

### 3.4 Visual diagram

**Figure 1:**
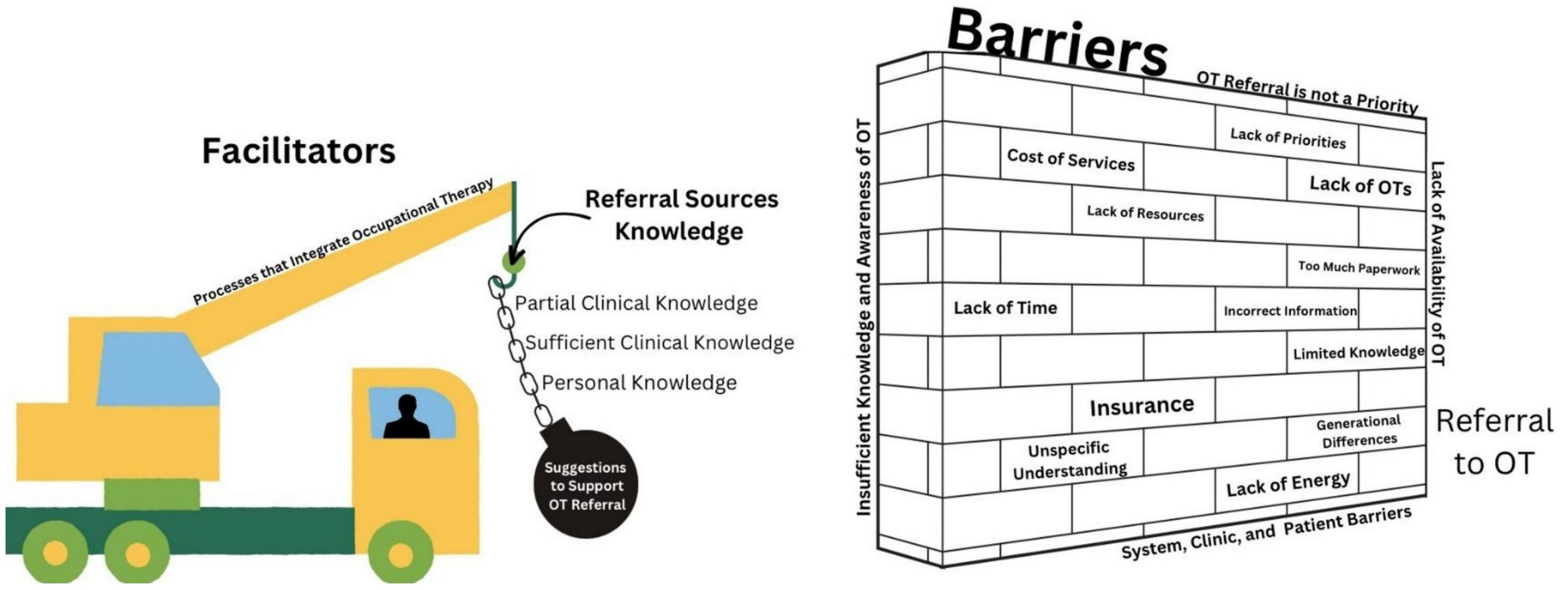
Logic diagram of facilitators, barriers, and strategies influencing OT referral in cancer care.

A visual diagram was developed (Figure 1) to capture the interconnections of the facilitators and barriers to OT referral in cancer care, as noted by participants. Although facilitators and barriers were prominent in this study, many challenges (illustrated in the figure as bricks) were interrelated and reinforced the main barrier themes to OT referral (sides of the wall). Overall, the diagram depicts the emergence of a theory from the data collected. It represents the power of leveraging facilitators to OT referral alongside the participants’ suggestions (chains and wrecking ball) to address barriers and improve referral to OT services in cancer care.

## 4 Discussion

This study provides an in-depth qualitative examination of facilitators and barriers to OT referrals for PLWBC from the perspective of referral sources in the United States, highlighting how referral practices shape access to supportive cancer care. The findings identified certain facilitators, particularly when OT services were embedded within interdisciplinary cancer-care teams, multiple barriers across individual-provider, organization/clinic, system, and patient levels continued to limit access to needed rehabilitation services. The characterization of OT as a “well-kept secret” encapsulates a central challenge in cancer-care delivery: Limited role clarity, competing clinical priorities, and structural constraints restrict OT utilization, even when services are available. Situating these findings alongside international evidence allows identification of healthcare-system–specific challenges and shared patterns influencing OT integration within cancer care.

Facilitators of OT referral included the integration of OT services into cancer-care pathways, regular collaboration between referral sources and OT practitioners, and referral sources’ knowledge of OT’s scope of practice. In the companion Israeli study, referral sources reported substantial gaps in linking cancer-related side effects to functional difficulties that would indicate the need for OT referral (9). In contrast, participants in the present (US) study demonstrated greater awareness of the relationships between occupational performance, cancer-related symptoms, and functional participation and more active referral to OT services within their organizations. Notably, some US participants described that directly integrating OT practitioners into cancer-care teams appeared to enhance understanding of OT’s role and support referrals. As in the Israeli study, referral sources’ personal experiences with OT—through interactions with family members or patients—also contributed to their knowledge and confidence regarding OT services (9).

When OT services are embedded within standard cancer care, opportunities for interdisciplinary interaction increase, enhancing referral sources’ understanding of OT’s role beyond impairment-focused interventions. These findings align with HPE and interprofessional collaboration literature, which emphasize that role clarification, interprofessional identity development, and recognition of profession-specific contributions support collaborative practice and referral decision-making (10–13, 22–25). They also align with OT-specific interprofessional education studies showing that direct exposure to OT can improve other health professionals’ understanding of OT roles and responsibilities and can facilitate OT integration into routine care (22, 26). When compared with the Israeli study, these findings highlight shared and context-specific facilitators of OT referral.

Despite these facilitators, participants identified several barriers to OT referral. Consistent with prior literature, the lack of OT availability and access emerged as a prominent organizational barrier. Previous studies similarly identified insufficient funding for OT positions and workforce limitations as barriers to oncology rehabilitation services (27). Calls for enhanced educational preparation of OT practitioners to support PLWBC further underscore the need to strengthen workforce capacity in cancer-care settings (2, 22). Beyond structural constraints, insufficient knowledge of OT persisted as a barrier, particularly in settings without OT practitioners on interdisciplinary teams. These findings are consistent with recent evidence on cancer rehabilitation, showing that access, referral, and participation are shaped by healthcare professionals’ knowledge, collaboration, resources, and referral pathways (15, 16, 28). The findings also suggest that limited role recognition is not simply an individual knowledge deficit, but a relational and organizational problem embedded within interprofessional practice (11, 12, 15, 28). Limited interactions reduce referral sources’ exposure to OT’s occupation-centered scope of practice, thereby constraining referral decision-making. This finding is consistent with recent narrative evidence (7) indicating that OT remains underutilized in oncology, partly because of limited awareness of its role and insufficient interdisciplinary collaboration. Consistent with this evidence and with expert consensus highlighting the importance of trained rehabilitation professionals within interdisciplinary cancer care (18), participants emphasized the need to better understand OT’s role and functional indicators warranting referral (23, 27, 29).

A distinctive finding in the US context was healthcare costs as a barrier to OT referrals, which did not emerge in the Israeli study. Cost-related concerns operated at the system and patient levels, influencing referral decisions and patient engagement alongside time constraints and treatment burden. Financial strain has been well documented as a source of distress among PLWBC, encompassing challenges related to medical bills, insurance navigation, and economic insecurity (18). These system-level barriers align with recent literature emphasizing that cancer rehabilitation access depends on coordinated referral pathways, service availability, reimbursement structures, and integration of rehabilitation into oncology workflows (15, 18, 28, 30). The current study extends prior work by demonstrating that financial considerations also shape not only patients’ uptake of services but also clinicians’ referral behaviors (8). These findings underscore the importance of clearly communicating the functional and participatory value of OT services, particularly in healthcare systems where cost considerations may discourage referrals despite clinical need.

Comparing findings from the US and Israeli studies reveals universal challenges and healthcare system-specific influences on OT access. Across contexts, shared challenges include limited knowledge of OT’s scope of practice, insufficient OT presence in oncology settings, busy clinic environments that deprioritize functional concerns, and the need for enhanced interdisciplinary collaboration. However, important differences also emerged. In the United States, healthcare costs, insurance complexity, fragmented care coordination, and variability in access based on insurance type and location were prominent barriers. In contrast, the Israeli study identified bureaucratic referral processes and limited community-based OT services as key obstacles, reflecting features of a centralized universal healthcare system. These findings underscore how financing and insurance structures directly influence clinicians’ supportive care referral decisions and highlight the role of health-system design in access to rehabilitation services. Shared challenges across healthcare systems point to opportunities for international collaboration in education, advocacy, and evidence dissemination.

Given the identified barriers, it was unsurprising that oncologists inconsistently prioritized referrals to OT. Limited role clarity, constrained availability of OT practitioners, busy clinical environments, and cost concerns collectively contribute to the low prioritization of OT referrals (26, 30). These findings underscore the need to increase knowledge of OT’s scope of practice and availability of OT practitioners within oncology clinics and community settings (8, 30, 31). As in the Israeli study (9), referral sources emphasized strengthening OT presence across cancer-care settings, including outpatient, home health, and infusion clinics, to address these challenges, reduce patient burden, and facilitate timely access to rehabilitation services. Participants also highlighted that integrating OT services into existing care workflows and coordinating OT and physical therapy appointments may enhance interdisciplinary collaboration and improve awareness of OT’s role. Participants’ recommendations are consistent with HPE and cancer rehabilitation literature, emphasizing the need for structured interprofessional learning, clearer role communication, and routine pathways that make rehabilitation professionals visible within oncology care (12, 15, 22, 32). Addressing OT’s characterization as a “well-kept secret” requires clearer communication and strategic approaches to increase visibility and support appropriate referral decision-making (5, 33, 34).

The similarities between the US and Israeli findings suggest that facilitators and barriers to OT referral reflect challenges beyond specific healthcare systems. These findings support the value of cross-national and cross-cultural research in better understanding the factors shaping OT integration in cancer care and in informing the development of internationally relevant strategies to improve access to rehabilitation services. Future studies should examine how identified multilevel barriers and facilitators can guide the design and evaluation of such strategies.

This study’s findings have important implications for supportive cancer-care delivery. Referral practices emerged as a key mechanism shaping access to supportive rehabilitation services across the cancer continuum. Delayed or absent referrals may result in unmet functional needs and increased patient burden. Embedding OT within interdisciplinary cancer-care models, such as survivorship care planning, navigation services, and infusion clinics, may facilitate earlier identification of functional concerns, improve care coordination, and reduce fragmentation. These recommendations are consistent with implementation evidence showing that integrating structured rehabilitation assessment into routine inpatient cancer care can improve awareness of rehabilitation needs, strengthen referral pathways, and support development of clinical pathways, referral systems, discharge coordination roles, and staff education (35). Strengthening referral pathways represents a critical opportunity to enhance equity, efficiency, and effectiveness of supportive cancer care across the survivorship continuum.

### 4.1 Strengths and limitations

This study has several strengths. First, the grounded theory approach and multiple-case study design enabled an in-depth exploration of referral processes across professional roles and practice settings. Second, the focus on referral sources, including nurses, physicians, and social workers, provides insight into the perspectives of professionals who often serve as gatekeepers for supportive rehabilitation services yet are less frequently centered in cancer rehabilitation research. Third, the study generated practical recommendations for improving OT referral pathways, including increasing OT presence in oncology settings, strengthening interdisciplinary collaboration, and clarifying OT’s role through targeted education. Methodological rigor was further supported by team-based coding, analytic memoing, member checking, and an external qualitative audit.

Several limitations of this study should also be considered. As qualitative research using focus groups, some participants may have felt less comfortable expressing dissenting views, and the use of remote videoconferencing may have limited rapport-building and observation of nonverbal communication. Recruitment through voluntary participation and professional networks may have introduced selection bias, as individuals with more experience or stronger opinions regarding OT may have been more likely to participate.

The sample was relatively small and consisted predominantly of nursing professionals, with limited representation of physicians, which may have influenced the range of perspectives captured. This imbalance is particularly important when interpreting physician-dependent referral barriers. Although participants across professional groups described referrals as highly dependent on physician initiation or endorsement, these findings primarily reflect interdisciplinary perceptions of physician-dependent referral processes rather than a comprehensive account of physicians’ own decision-making. Therefore, findings on physician-dependent referral barriers should be interpreted with caution and examined further in future studies with greater physician representation. Furthermore, although low physician enrollment was noted during data collection, the research team did not pursue secondary recruitment adjustments or submit an IRB addendum to deploy enhanced, targeted recruitment strategies for this sub-population. Future research should use targeted recruitment frameworks or dedicated institutional protocol amendments to ensure a more balanced distribution of voices across the oncology multidisciplinary care team. Participants were recruited from a limited number of states, potentially restricting transferability to settings with different healthcare structures, insurance models, or referral pathways. Further, only referral sources were included; thus, findings may reflect clinicians’ perspectives rather than patients’ experiences. Finally, the study examined perceived barriers and facilitators to OT referral without assessing referral rates or objectively measuring referral behaviors, which future research should address.

## 5 Conclusion

Referral sources in the US study demonstrated a relatively deeper understanding of OT’s scope of practice than in the Israeli study; however, OT services were not consistently prioritized in cancer care in either case. Persistent barriers, including limited availability of OT services, insufficient role clarity, competing clinical priorities, and cost-related reimbursement constraints, continued to restrict access to rehabilitation.

Despite these challenges, participants identified actionable strategies to improve OT integration. They emphasized embedding OT practitioners in interdisciplinary cancer-care teams; expanding service delivery to outpatient, community, and home health settings; and increasing OT presence in high-contact environments, such as infusion clinics.

This international collaboration highlights shared challenges across healthcare systems and underscores the importance of improving visibility and communication of OT’s occupation-centered contribution. Strengthening referral pathways to OT represents a critical opportunity to enhance the equity, coordination, and effectiveness of supportive cancer care across the survivorship continuum.

## Data Availability

The de-identified data supporting the conclusions of this article are available from the corresponding author upon reasonable request, subject to ethical and confidentiality considerations.
