# Supplementary material for: A “well-kept secret”: facilitators and barriers to occupational therapy referral as a component of supportive cancer care in the United States
Source: Front Med (Lausanne). 2026 Aug 5;13:1799055. doi: 10.3389/fmed.2026.1799055 (PMC13486235; doi:10.3389/fmed.2026.1799055)
Supplement: Supplementary file 1 [file Supplementary_file_2.pdf]

A "Well-Kept Secret": Facilitators and Barriers to Occupational Therapy Referral as a Component of Supportive Cancer Care in the United States

Authors: Khawla Loubani*, Rachel Graves, Taylor Brown, Cara Murphy, Alexandria Gilley, Olivia Witteborg, Korrin Schalhamer, Morgan Jones, Katie M. Polo
Email: khawlalo@bgu.ac.il

“Hello and welcome everyone. My name is Taylor/Lexa and I am an OTD student working with Dr. Katie Polo on this research project. Thank you all for being here this morning and being willing to participate in our research. As you know, the purpose of this study is to explore the perspectives of healthcare professionals who work with cancer patients regarding the role of OT and what current barriers and facilitators for referrals to OT exist during and following cancer treatment. I want to remind you that being here today indicates your consent to participate in our research and that all information or answers shared here today will be deidentified in the transcripts. Participation is voluntary— please feel no pressure to answer questions you do not want to. In addition to myself, there are some other research team members on this call with us all of whom are also OTD students at UIndy. They will be muted in the background taking notes and making sure the technology runs smoothly. Before we get started, I would like to invite them to introduce themselves with their name and role.

research team intros

Thank you, research team. For the interview they will be muted with their cameras off, but they will rejoin us at the end for a summary. Are there any questions? If not, we will get started by having each of you introduce yourself by stating your name, your occupational title, and where you live. For example, my name is Taylor/Lexa, I am an OTD student, and I live and study in Indianapolis. I will ask (insert participant name) to start.

when intros are finished

Wonderful, thank you. It’s so nice to meet you all. I will now jump into our questions, which I anticipate will take about 45 minutes to complete."

A "Well-Kept Secret": Facilitators and Barriers to Occupational Therapy Referral as a Component of Supportive Cancer Care in the United States

Authors: Khawla Loubani*, Rachel Graves, Taylor Brown, Cara Murphy, Alexandria Gilley, Olivia Witteborg, Korrin Schalhamer, Morgan Jones, Katie M. Polo
Email: khawlalo@bgu.ac.il

Table 2.

Interview Guide

Interview Guide

1) To explore the perspectives of healthcare professionals regarding the role of occupational therapy to the care of cancer patients.

1a. What do you know about occupational therapy in general?

1b. What do you know regarding occupational therapy's role in oncology?

- *"I have not yet, but could see how I would." What are some situations where you could see OT being used in your work?*

2) To explore healthcare professionals' knowledge of the need for occupational therapy services for cancer patients.

2a. How does cancer affect your patient's daily functioning and daily activities?

2b. What are the signs and/or indicators to refer your patients to occupational therapy?

2c. What limitations in your patients' daily functioning might prompt you to refer to occupational therapy?

3) To understand the current barriers to occupational therapy referrals from healthcare professionals' perspectives.

3a. What are the reasons that cancer patients are not referred to occupational therapy?

4) To explore what is needed to optimize referrals to occupational therapy.

4a. What do you think is needed to optimize referral of your patients to occupational therapy?

A "Well-Kept Secret": Facilitators and Barriers to Occupational Therapy Referral as a Component of Supportive Cancer Care in the United States
Authors: Khawla Loubani*, Rachel Graves, Taylor Brown, Cara Murphy, Alexandria Gilley, Olivia Witteborg, Korrin Schalhamer, Morgan Jones, Katie M. Polo
Email: khawlalo@bgu.ac.il

National data base -tertiary and cancer centers vs rural

Is there anything else you would like to share?

Wrap-Up

Right now, our note takers are going to give a quick summary of what we talked about today.

Note takers: Summary. After hearing the summary, is there anything you would like to add or anything you would recommend we change?

Before we end the meeting, we would love if you could share this project with your colleagues, feel free to send them to Korrin Schalhamer, your original contact. If you are local, we are willing to set up in-person meetings if that would be more convenient. Thank you again, and have a good day!
