# Supplementary material for: A “well-kept secret”: facilitators and barriers to occupational therapy referral as a component of supportive cancer care in the United States
Source: Front Med (Lausanne). 2026 Aug 5;13:1799055. doi: 10.3389/fmed.2026.1799055 (PMC13486235; doi:10.3389/fmed.2026.1799055)
Supplement: Supplementary file 2 [file Supplementary_file_1.docx]

**Supplementary Material 1. Demographic and Professional Background Questionnaire**

Please complete the following demographic and professional background questionnaire before participating in the focus group.

1. Age: ______________________

2. Gender:

☐ Woman

☐ Man

☐ Prefer not to answer

3. Highest education level completed:

☐ Associate degree

☐ Bachelor’s degree

☐ Master’s degree

☐ Doctoral degree

☐ Professional degree, please specify: ______________________

☐ Other, please specify: ______________________

4. Profession:

☐ Nurse

☐ Nurse navigator

☐ Physician/oncologist

☐ Physician assistant

☐ Social worker

☐ Other, please specify: ______________________

5. Specialty or area of practice: ______________________

6. State of practice: ______________________

7. Practice setting:

☐ Hospital

☐ Community cancer center

☐ Cancer clinic

☐ Academic medical center

☐ Other, please specify: ______________________

8. Years working in oncology or cancer care: ______________________

9. Workplace/unit/department: ______________________

11. Current job title: ______________________

12. Do you actively refer your clients/patients to occupational therapy?

☐ Yes

☐ No

☐ Not applicable

☐ Other, please specify: ______________________

13. In your workplace role, are you able to directly collaborate with occupational therapists?

☐ Yes

☐ No

☐ Not sure

☐ Not applicable

14. How often are you able to directly collaborate with occupational therapists?

☐ Never

☐ Rarely

☐ Sometimes

☐ Often

☐ Very often

☐ Not applicable
